# Editorial: The Role of Opioid Receptors in Immune System Function

**DOI:** 10.3389/fimmu.2021.832292

**Published:** 2022-01-10

**Authors:** Thomas J. Rogers, Sabita Roy

**Affiliations:** ^1^ Temple University, Philadelphia, PA, United States; ^2^ University of Miami Health System, Miami, FL, United States

**Keywords:** opioid, opioid receptor, HIV, inflammation, neuroinflammation

It has long been appreciated that opioids, such as morphine, exhibit anti-inflammatory activity. This led early on to the speculation that either cells of the nervous system influence the activities of the immune system, or that cells of the immune system express opioid receptors. Early studies with hematopoietic cells to determine the presence of opioid receptors were difficult to evaluate because these cells do not express the opioid receptors at levels which are expressed in the CNS. There are four opioid receptor types expressed by neuronal cells, µ (MOP), δ (DOP), κ (KOP), and a related receptor for the peptide nociception (NOP). Reports during the 1990s established that hematopoietic cells do, in fact, express each of the opioid receptor types, and the gene products which are expressed by these cells are identical to those expressed by cells of the nervous system (1–5). Indeed, the opioid receptors are widely expressed within both the central and peripheral nervous systems, and by both lymphoid and myeloid cells of the immune system.

It has become clear from a large volume of data that while the opioids have immunosuppressive activity for antibody responses, natural killer cell activity, phagocytic capacity, and other responses based on both *in vivo* and *in vitro* studies, carried out with both human and laboratory animals [reviewed in (6, 7)]. A large volume of literature shows that opioid drug abuse leads to greater susceptibility to viral and bacterial infections. It is particularly notable that intravenous administration of heroin represents a primary route of HIV infection (approximately 10% of cases worldwide). The resulting pathology following the combination of HIV infection and chronic opioids are particularly evident in organ systems including the CNS and the lungs. The opiate abuse promotes the progression of the pathology associated with HIV infection, and there is evidence that this is due to the combined impact of the chronic opiate exposure and the inflammatory effects of the HIV infection (8). Chronic opioid abuse, particularly with chronic intravenous self-administration, activates peripheral and central mu opioid receptors (MOR), leading to an inflammatory reaction in both the brain and lungs. Moreover, chronic opioid use in the HIV-infected individual exacerbates the inflammation in the brain, as well as inflammation in the lungs resulting in obstructive lung disease.

The review article from Machelska and Celik serves as an excellent introduction to this series of articles by providing an overview of the expression of opioid receptors on cells of the immune system, and the biochemical consequences of the activation of these receptors. The article goes on to show that the cells of the immune system also serve as a source of opioid peptides that serve as agonists for the excitation of opioid receptors in the periphery. Based on all of this, the article also demonstrates the relationship between the expression of the opioid agonists and receptors on the regulation of pain responses.

The highly viewed (>21,000 views) article by Eisenstein reviews the results from investigation of the effects of opioids on immune responses, which was fueled by an interest in the intersection of intravenous heroin abuse in HIV-infected individuals. While the influence of opiates like heroin is predominantly to activate MOR, and induce immunosuppression, it was not initially clear that this would lead to an accelerate HIV infection. Much of the work over the last several decades has been directed toward an understanding of the mechanisms that are responsible for the neuroinflammation and pathology that occurs in the HIV-infected opiate abuser.

The review article by Franchi et al. analyzes the available literature on the immunomodulation induced by various opioid drugs in both human and experimental animal models. It reviewing The results of these studies must be carefully evaluated since experiments are often conducted using a wide range of opioid doses. Moreover, some published work may involve analysis of a relatively small number of participants, so drawing general conclusions regarding capacity of opioids to induce immunosuppression may be an oversimplification of the activities of all opioid drugs.

The intersection between the current opioid abuse epidemic and the HIV/AIDS pandemic is important for our understanding of the role of opioids in the function of the immune system. The original article by Meng et al. shows how the combination of HIV infection and opioid use can lead to intestinal barrier dysfunction and microbial dislocation. This can lead to a significant alteration in the gut microbiome, as well as substantial consequences in the status of the immune response in HIV-infected patients.

From a different perspective in the review by Wang et al., the authors demonstrate that both morphine abrupt withdrawal and precipitated withdrawal enhance the susceptibility of macrophages to HIV infection, and this leads to the activation of HIV replication in latently infected myeloid cells. Very significantly this then leads to a reduction in the expression of several intracellular HIV inhibitory factors. These findings important because they suggest that a major influence of the opioid use is to interfere with intracellular anti-HIV immunity, and as a consequence, promote HIV infection and persistence in macrophages.

The Murphy et al. article reviews the complex basis for the participation of opioids in the neuroinflammation associated with HIV infection. The article goes on to provide an explanation for the biochemical and molecular processes that are likely to participate in the neuropathogenesis associated with HIV infection and opioid abuse. Finally, these authors extend their review to include novel technologies and potential therapies for these patients, in the context of currently employed antiretroviral drugs.

The original article by Dave et al. provides a description of studies conducted with simian human immunodeficiency virus (SHIV) carried out in rhesus macaques. The article shows that the administration of morphine to SHIV-infected animals leads to a significant alteration in the expression of proinflammatory cytokines and chemokines, and this may have consequences for the neuropathogenesis induced by the viral infection.

Finally, the review by Rogers describes the cross-talk between opioid and chemokine receptors, and the consequences for the functional activity of these receptors. Several laboratories have reported that the activation of opioid receptors results in the regulation of the expression of several pro-inflammatory chemokines and chemokine receptors. However, beyond this, the activation of opioid receptors signals the desensitization of certain pro-inflammatory chemokine receptors. The reverse process can also occur, and this can have a significant impact both for the recruitment of leukocytes, as well as the sensation of pain, at the site of an inflammatory response.

## Author Contributions

Both authors conceived, designed, and wrote the manuscript. Both authors have read and approved the final version of the manuscript.

## Funding

This work was supported by National Institutes of Health grants RO1 DA 040619, RO1 DA 049745, P30 DA 13429, awarded to TR and R01DA044582, R01DA043252, R01DA050542, R01DA050542, R01DK117576, and T32DA045734 awarded to SR.

## Conflict of Interest

The authors declare that the research was conducted in the absence of any commercial or financial relationships that could be construed as a potential conflict of interest.

## Publisher’s Note

All claims expressed in this article are solely those of the authors and do not necessarily represent those of their affiliated organizations, or those of the publisher, the editors and the reviewers. Any product that may be evaluated in this article, or claim that may be made by its manufacturer, is not guaranteed or endorsed by the publisher.
